# Reproducibility of short axis slice locations in longitudinal cardiovascular magnetic resonance (CMR) studies

**DOI:** 10.1186/1532-429X-16-S1-P228

**Published:** 2014-01-16

**Authors:** Christopher Kelly, Vicente Grau, Stefan Neubauer, Robin Choudhury, Erica Dall'Armellina

**Affiliations:** 1 Department of Cardiovascular Medicine, University of Oxford Centre for Clinical Magnetic Resonance Research, Oxford, UK; 2 Department of Engineering Science, Institute of Biomedical Engineering, Oxford, UK; 3 Department of Cardiovascular Medicine, Acute Vascular Imaging Centre (AVIC), Oxford, UK

## Background

Longitudinal dynamic changes of myocardial ischemic injury determine left ventricular (LV) remodelling. Using conventional clinical cardiovascular magnetic resonance (CMR) imaging, the registration of short axis (SA) images for comparison of imaging findings at different time points is aided by the application of the same protocol to consistently identify the atrio-ventricular (AV) groove and SA planes. We hypothesise that, discrepancies in the selection of short and long axis planes lead to significant differences in location and angle between SA slices manually identified as corresponding in two studies of the same patient.

## Methods

Six ST elevation myocardial infarction (STEMI) patients underwent 3T CMR at 24 hrs and 6 months post primary percutaneous coronary intervention (PPCI). The CMR protocol included functional steady state free precession (SSFP) imaging of long axis (LA) slices and contiguous short axis (SA) slices (8 mm thick with 2 mm gap) parallel to the AV groove with full LV coverage. A very experienced operator performed the scans. For each subject, 2D registration of a single selected LA slice, acquired at the two visits, was performed using a point-based approach. The corresponding 3D transformation in physical space was calculated using slice locations and angles in Dicom tags. Two SA slices from each visit, were selected in the mid-cavity area, considered through visual inspection to provide the best match between the studies. The calculated 3D transformation was then applied to the SA slice from the first visit. The alignment error between the SA slices was quantified in terms of their angular difference and offset (mm) and averaged over a manually selected region of interest (ROI) containing approximately the area of the heart.

## Results

**Figure 1:**
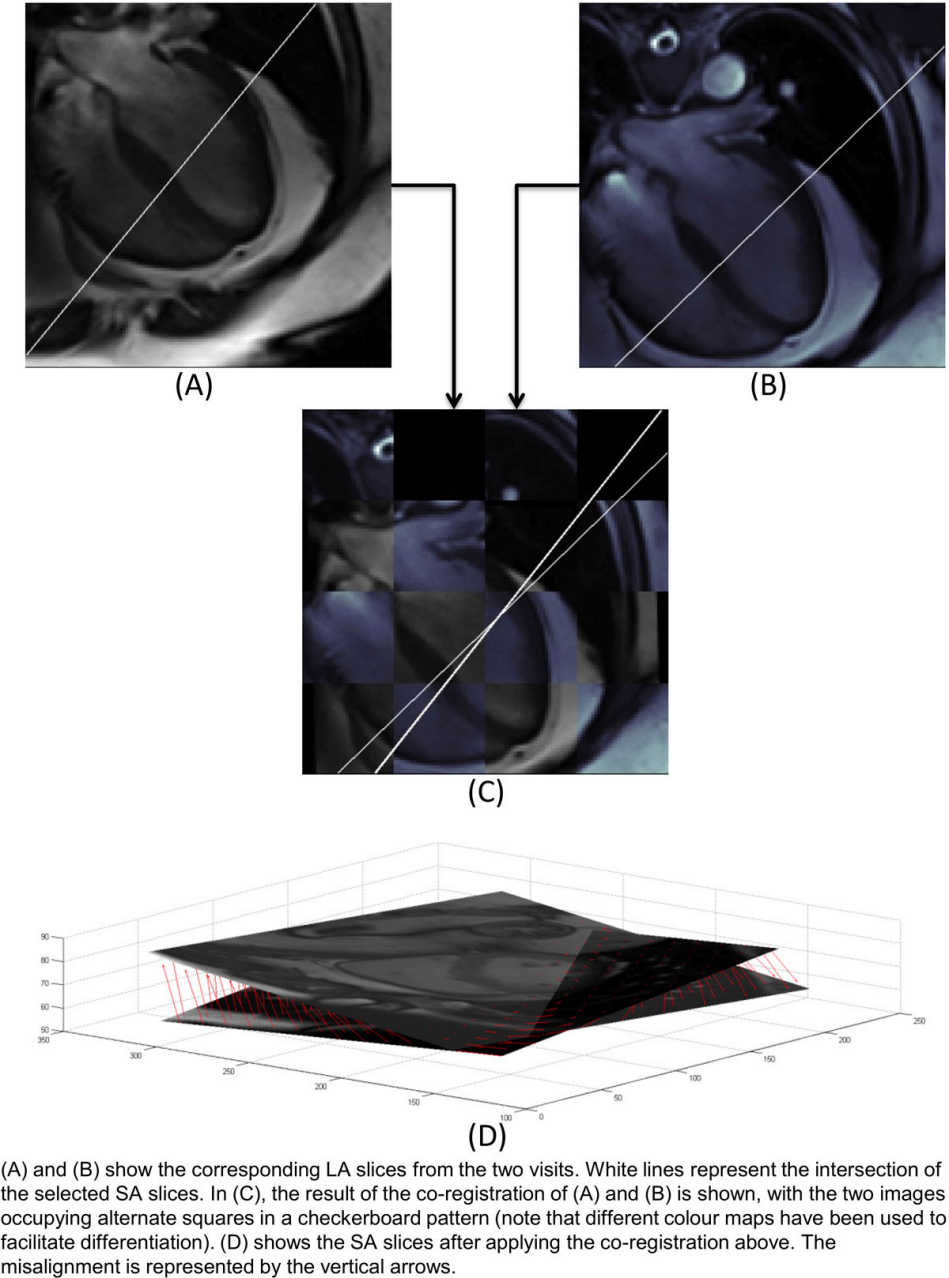
**(A) and (B) show the corresponding LA slices from the two visits**. White lines represent the intersection of the selected SA slices. In (C), the result of the co-registration of (A) and (B) is shown, with the two images occupying alternate squares in a checkerboard pattern (note that different colour maps have been used to facilitate diffentiation). (D) shows the SA slices after applying the co-registration above. The misalignment is represented by the vertical arrows.

In all patients, there was a substantial misalignment between the SA slices. The angular difference measured between the SA slices was 8.3 degrees ± 7.5 degrees (mean ± SD). The average offset for the heart ROI was 6.5 mm ± 6.5 mm, which is greater than half the inter-slice spacing of 10 mm. (Figure 1)

## Conclusions

Conventional manual registration of CMR images acquired at different time points, leads to significant misalignment. This may affect significantly the post-processing of in-plane changes of myocardial injury especially for lesions with irregular shapes or sizes similar to the calculated error values. Further investigations will be needed to quantify the clinical significance of these findings.

## Funding

Oxford BIomedical Research Centre EPSRC Doctoral Training Award BBSRC and the British Heart Foundation.

